# Predictive and interpretable models via the stacked elastic net

**DOI:** 10.1093/bioinformatics/btaa535

**Published:** 2020-05-21

**Authors:** Armin Rauschenberger, Enrico Glaab, Mark A van de Wiel

**Affiliations:** Luxembourg Centre for Systems Biomedicine (LCSB), University of Luxembourg, 4362 Esch-sur-Alzette, Luxembourg; Department of Epidemiology and Data Science, Amsterdam UMC, 1081 HV Amsterdam, The Netherlands; Luxembourg Centre for Systems Biomedicine (LCSB), University of Luxembourg, 4362 Esch-sur-Alzette, Luxembourg; Department of Epidemiology and Data Science, Amsterdam UMC, 1081 HV Amsterdam, The Netherlands; MRC Biostatistics Unit, University of Cambridge, CB2 0SR Cambridge, UK

## Abstract

**Motivation:**

Machine learning in the biomedical sciences should ideally provide predictive and interpretable models. When predicting outcomes from clinical or molecular features, applied researchers often want to know which features have effects, whether these effects are positive or negative and how strong these effects are. Regression analysis includes this information in the coefficients but typically renders less predictive models than more advanced machine learning techniques.

**Results:**

Here, we propose an interpretable meta-learning approach for high-dimensional regression. The elastic net provides a compromise between estimating weak effects for many features and strong effects for some features. It has a mixing parameter to weight between ridge and lasso regularization. Instead of selecting one weighting by tuning, we combine multiple weightings by stacking. We do this in a way that increases predictivity without sacrificing interpretability.

**Availability and implementation:**

The R package starnet is available on GitHub (https://github.com/rauschenberger/starnet) and CRAN (https://CRAN.R-project.org/package=starnet).

## 1 Introduction

**Fig. 1.**
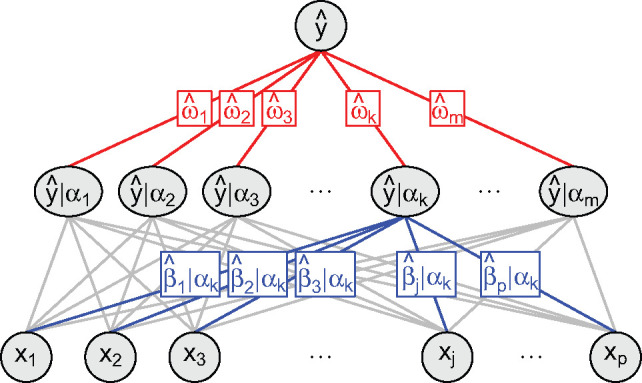
Stacked elastic net. After predicting the outcome from the features given an elastic net mixing parameter (bottom), we combine the predictions from multiple elastic net mixing parameters (top)

High-dimensional regression requires regularization. The elastic net (Zou and Hastie, 2005) generalizes ridge $\left(L_{2}\right)$ and lasso $\left(L_{1}\right)$ regularization, and overcomes some of their shortcomings. Adapting the sparsity of the model to the sparsity of the signal, it often improves predictions. One issue with the elastic net is that it has two tuning parameters: either two regularization parameters $\lambda_{1}$ and $\lambda_{2}$ for ridge and lasso, or one regularization parameter λ and one mixing parameter α for moderating between ridge and lasso. Tuning both α and λ is notoriously hard due to the flat cross-validated likelihood landscape (van de Wiel *et al.*, 2019). Alternatively, fixing α close to the lasso might be a good solution, because this introduces stability (Friedman *et al.*, 2010). As an alternative to tuning or fixing α, we propose to combine multiple values of α, using stacked generalization (Wolpert, 1992). Each α renders one model with one estimated effect for each feature. Instead of selecting one α for making predictions, stacking combines the predictions from multiple α (Fig. 1). The resulting ensemble model (multiple α) might be more predictive than any of the constituent models (single α) but is less interpretable due to multiple effects for each feature (one for each α). Rather than combining the predicted values from the base learners, we propose to combine their linear predictors. This allows us to rewrite the complex model (with multiple effects for each feature) as a simple model (with one effect for each feature). The stacked elastic net thereby increases predictivity while maintaining the interpretability of the regression coefficients. Furthermore, feature selection is possible after model fitting (Hahn and Carvalho, 2015). In the following, we introduce the stacked elastic net, analyse simulated and experimental high-dimensional data and discuss possible extensions.

## 2 Materials and methods

### 2.1 Base learners

The data consist of one outcome and *p* features for *n* samples, possibly in a high-dimensional setting (p≫n). For example, the outcome might represent a clinical variable, and the features might represent molecular data. Let the n×1 vector ***y*** denote the outcome, and let the n×p matrix ***X*** denote the features. We index samples by i∈{1,…,n} and features by j∈{1,…,p}. In the generalized linear model framework, we have

$$E[y_{i}]=h^{-1}\left(\beta_{0}+\sum_{j=1}^{p}\beta_{j}X_{ij}\right),$$

where h(·) is a link function, $\beta_{0}$ is the unknown intercept and $\beta ={\left(\beta_{1},\ldots ,\beta_{p}\right)}^{⊺}$ are the unknown slopes. Penalized maximum-likelihood estimation involves determining

$$\{{\hat{\beta }}_{0},\hat{\beta }\}=\underset{\beta_{0},\beta }{\text{argmax}}\{L(y;\beta_{0},\beta )-\rho (\lambda ,\alpha ;\beta )\},$$

where $L(y;\beta_{0},\beta )$ is the likelihood, and ρ(λ,α;β) is the elastic net penalty. The likelihood depends on the type of regression model (e.g. Gaussian, binomial or Poisson), and the penalty function is

$$\rho (\lambda ,\alpha ;\beta )=\lambda \sum_{j=1}^{p}\left(\frac{1-\alpha }{2}\beta_{j}^{2}+\alpha |\beta_{j}|\right),$$

where λ is the regularization parameter (λ≥0), and α is the elastic net mixing parameter (0≤α≤1). The limits correspond to ridge (α=0) and lasso (α=1) regularization. We consider *m* different values for α, which are equally spaced in the unit interval and indexed by k∈{1,…,m}. For each $\alpha_{k}$, we use 10-fold cross-validation for tuning $\lambda_{k}$. We consider an exponentially decreasing sequence of values for $\lambda_{k}$, starting with the intercept-only model $\left(\lambda_{k}\to \infty \right)$ and stopping with the (almost) unpenalized model $\left(\lambda_{k}\to 0\right)$. In short, we select the optimal $\lambda_{k}^{*}$ for each $\alpha_{k}$. We retain the corresponding cross-validated linear predictors in the n×m matrix ${\hat{H}}^{\left(\text{cv}\right)}$.

### 2.2 Meta learner

We then regress the outcome on the cross-validated linear predictors:

$$E[y_{i}]=h^{-1}\left(\omega_{0}+\sum_{k=1}^{m}\omega_{k}{\hat{H}}_{ik}^{\left(\text{cv}\right)}\right)\;,$$

where $\omega_{0}$ is the unknown intercept, and $\omega ={\left(\omega_{1},\ldots ,\omega_{m}\right)}^{⊺}$ are the unknown slopes. The intercept might allow the meta learner to reduce systematic errors from strongl*y* correlated base learners. Since the slopes are weights, we constrain them to the unit interval, i.e. $0\leq \omega_{k}\leq 1$ for all k∈{1,…,m}. They weight the linear predictors from the different elastic net mixing parameters. Penalized conditional maximum-likelihood estimation involves determining

$$\{{\hat{\omega }}_{0},\hat{\omega }\}=\underset{\omega_{0},\omega }{\text{argmax}}\{L(y;\omega_{0},\omega )-\rho (\lambda ;\omega )\}\;,$$

where $L(y;\omega_{0},\omega )$ is the likelihood conditional on ${\hat{H}}^{\left(\text{cv}\right)}$, and ρ(λ;ω) is the lasso penalty

$$\rho (\lambda ;\omega )=\lambda \sum_{k=1}^{m}\left|\omega_{k}\right|\;.$$

Using the same cross-validation folds as for the base learners, we select the optimal regularization parameter $\lambda^{*}$ for the meta learner. Accordingly, in the two consecutive cross-validation loops, we use the same training sets for estimating the base and meta parameters ($\beta_{0}$ and β given $\alpha_{k}$ for all *k*; $\omega_{0}$ and ω), and the same validation sets for tuning the base and meta hyperparameters (${\text{λ}}_{k}$ for all *k*; λ).

The tuned elastic net is a special case of the stacked elastic net: if the intercept equals zero ($\omega_{0}=0$), one weight equals one ($\omega_{k}=1$), and all other weights equal zero ($\omega_{l\neq k}=0$), the meta learner simply selects one mixing parameter ($\alpha_{k}$). In a broader sense, van der Laan *et al.* (2007) distinguish between *cross-validation selection* and *super-learning*, which consist of selecting one or combining multiple base learners, respectively.

### 2.3 Combination

Given the cross-validated parameters $\lambda^{*}={\left(\lambda_{1}^{*},\ldots ,\lambda_{m}^{*}\right)}^{⊺}$ and $\lambda^{*}$, we refit the base and meta learners to all folds. For the base learners, let the 1×m vector ${\hat{\beta }}_{0}$ and the p×m matrix $\hat{\beta }$ denote the estimated intercepts and slopes, respectively. For the meta learner, the estimates are ${\hat{\omega }}_{0}$ and $\hat{\omega }={\left({\hat{\omega }}_{1},\ldots ,{\hat{\omega }}_{m}\right)}^{⊺}$. We then use the estimates from the base and meta learners to predict the outcome of previously unseen samples.

If sample *i* has the feature vector $X_{i○}={\left(X_{i1},\ldots ,X_{ip}\right)}^{⊺}$, base learner *k* returns the linear predictor 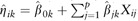. The meta learner combines the linear predictors from all base learners: 
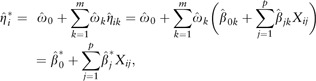
 where 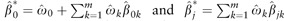. Since the *stacked* linear predictor is a function of *pooled* estimates, we perform stacking without loss of interpretability. For each feature, the corresponding pooled estimate represents the estimated effect on the outcome. Due to ridge regularization in one of the base learners, however, all pooled estimates might be different from zero. Stacking worsens the variable selection property of the elastic net, but we still have the option to select variables after model fitting (see below).

### 2.4 Extension

Decoupling shrinkage and selection (Hahn and Carvalho, 2015) allows us to perform feature selection after model fitting. The idea is to approximate the fitted linear predictor ${\hat{\eta }}^{*}=X{\hat{\beta }}^{*}$ by $X\hat{\gamma }$, where ${\hat{\beta }}^{*}$ is dense but $\hat{\gamma }$ is sparse. Instead of including many features 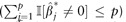, we only want to include some features ($\sum_{j=1}^{p}I[{\hat{\gamma }}_{j}\neq 0]\ll p$). This can be achieved by regressing the fitted linear predictor on the features and estimating a sparse model: 
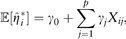
 where γ_0_ is the unknown intercept, and $\gamma ={\left(\gamma_{1},\ldots ,\gamma_{p}\right)}^{⊺}$ are the unknown slopes. Penalized maximum-likelihood estimation involves determining 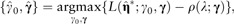 where $L({\hat{\eta }}^{*};\gamma_{0},\gamma )$ is the Gaussian likelihood, and ρ(λ;γ) is the adaptive lasso penalty (Zou, 2006) 
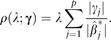


The absolute values of the dense estimates (${\hat{\beta }}^{*}$) operate as weights for the sparse estimates ($\hat{\gamma }$). As λ increases from 0 to ∞, the number of non-zero coefficients decreases from min(n,p) to 0. We can cross-validate λ, or adjust λ in order that the model includes a specific number of non-zero coefficients (e.g. $\sum_{j=1}^{p}I[{\hat{\gamma }}_{j}\neq 0]=10$). We expect this approximation to work well when the pooled estimates are relatively sparse, i.e. include few values far from zero and many values close to zero. Such a situation is fairly natural for the stacked elastic net because it pools mainly sparse and strongly correlated models. Nevertheless, *post-hoc* feature selection might significantly decrease the predictive performance of the stacked elastic net, and should therefore be used with caution.

## 3 Simulation

### 3.1 Prediction accuracy

To examine the predictive performance of the stacked elastic net, we conducted a simulation study. We compared ridge, lasso, tuned elastic net and stacked elastic net regularization.

In three different scenarios, we repeatedly simulated high-dimensional data. In each iteration, we sampled several *n*-dimensional vectors from the standard Gaussian distribution, namely three signal variables $\left(z_{1},z_{2},z_{3}\right)$ and *p* noise variables $\left(\varepsilon_{1},\ldots ,\varepsilon_{p}\right)$. We constructed the outcome from the signal variables, and the features from the signal and noise variables. In all scenarios, the *n*-dimensional outcome vector equals the sum of the three signal variables $\left(y=z_{1}+z_{2}+z_{3}\right)$. The *n * ×*  p* feature matrix ***X***, however, depends on the scenario (Table 1). Let $x_{j}$ denote the jth column of ***X***, for any *j* in {1,…,p}. Each feature equals a weighted sum of one signal variable and one noise variable: $x_{j}=\sqrt{\pi }z_{l}+\sqrt{1-\pi }\varepsilon_{j}$, where the weight π is in the unit interval, and the index *l* equals 1 or 2. The weight π determines whether the feature is weakly (π=0.1), moderately (π=0.5) or strongly (π=0.9) correlated with the signal variable, and consequently weakly, moderately or highly predictive of the outcome. In the first scenario, one feature is strongly correlated with $z_{1}$, and another feature is strongly correlated with $z_{2}$. In the second scenario, 50% of the features are weakly correlated with $z_{1}$, and the other 50% are weakly correlated with $z_{2}$. And in the third scenario, 5% of the features are moderately correlated with $z_{1}$, and another 5% of the features are moderately correlated with $z_{2}$. The weighting ensures that all features have unit variance: $\text{Var}(x_{j})=\pi \text{Var}(z_{l})+(1-\pi )\text{Var}(\varepsilon_{j})=1$ because $\text{Var}(z_{l})=1,\;\text{Var}(\varepsilon_{j})=1$ and $\text{Cov}(z_{l},\varepsilon_{j})=0$.

**Table 1.** Scenarios for constructing features $\left(x_{1},\ldots ,x_{500}\right)$ from signal $\left(z_{1},z_{2},z_{3}\right)$ and noise $\left(\epsilon_{1},\ldots ,\epsilon_{500}\right)$

	signal + noise	noise	signal + noise
(1)	$x_{j}=\sqrt{0.9}z_{1}+\sqrt{0.1}\epsilon_{j}$ j=1	$\begin{array}{c} x_{j}=\epsilon_{j}\\ \forall j\in \{2,\ldots ,499\} \end{array}$	$\begin{array}{c} x_{j}=\sqrt{0.9}z_{2}+\sqrt{0.1}\epsilon_{j}\\ j=500 \end{array}$
(2)	$\begin{array}{c} x_{j}=\sqrt{0.1}z_{1}+\sqrt{0.9}\epsilon_{j}\\ \forall j\in \{1,\ldots ,250\} \end{array}$	–	$\begin{array}{c} x_{j}=\sqrt{0.1}z_{2}+\sqrt{0.9}\epsilon_{j}\\ \forall j\in \{251,\ldots ,500\} \end{array}$
(3)	$\begin{array}{c} x_{j}=\sqrt{0.5}z_{1}+\sqrt{0.5}\epsilon_{j}\\ \forall j\in \{1,\ldots ,25\} \end{array}$	$\begin{array}{c} x_{j}=\epsilon_{j}\\ \forall j\in \{26,\ldots ,475\} \end{array}$	$\begin{array}{c} x_{j}=\sqrt{0.5}z_{2}+\sqrt{0.5}\epsilon_{j}\\ \forall j\in \{476,\ldots ,500\} \end{array}$

**Fig. 2.**
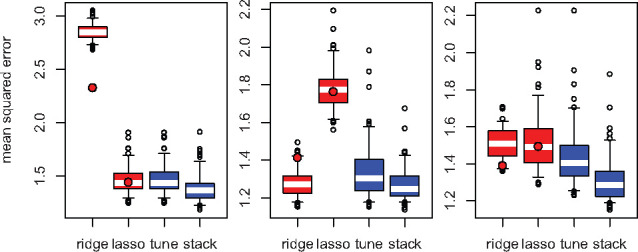
Out-of-sample mean squared error in the first (left), second (centre) and third (right) scenarios. The filled circles indicate the medians from the ridge-like (α=0.05) and lasso-like (α=0.95) elastic net. (The boxes show the interquartile ranges, and the whiskers show the ranges from the 5th to the 95th percentiles)

In each scenario, we simulated the outcome (n×1 vector ***y***) and the features (*n  *× * p* matrix ***X***) each 100 times, where n=10 000 and *p *=* *500. We assessed the predictive performance using 100 samples for training and validation (internal 10-fold cross-validation) and 9900 samples for testing (hold out). Figure 2 shows the mean squared error for the test set under different flavours of elastic net regularization (ridge, lasso, tuning, stacking). These out-of-sample errors are estimates of the predictive performance on previously unseen data, with lower values indicating better predictions. Lasso outperforms ridge if the signal is sparse (1st scenario), but ridge outperforms lasso if the signal is dense (2nd scenario). Approaching the performance of the optimal elastic net mixing parameter, tuning is slightly worse than lasso in the sparse case (1st scenario), slightly worse than ridge in the dense case (2nd scenario), or better than both in the intermediate case (3rd scenario). We observe that stacking outperforms tuning in all three scenarios. Stacking is even slightly better than lasso in the sparse case and slightly better than ridge in the dense case. The most important gains relative to the best competitor occur in the intermediate case. In the three scenarios, stacking is the best approach in 79%, 67% and 88% of the iterations, respectively.

Next, we tested whether stacking leads to significantly better predictions than ridge, lasso and tuning. For this purpose, we calculated the pairwise differences in out-of-sample mean squared error, applied the two-sided Wilcoxon signed-rank test and used the Bonferroni-adjusted 5% significance level (*P*-value ≤0.05/9). Stacking significantly outperforms tuning in all three scenarios. Moreover, stacking is significantly better than ridge and lasso, but not significantly different from ridge if the signal is dense (2nd scenario). In practice, we often do not know whether ridge or lasso is more suitable for the data at hand. An advantage of the elastic net is that it automatically adapts to the sparsity level of the signal.

For comparison, we also examined the elastic net with the fixed mixing parameters α=0.05 (close to ridge) and α=0.95 (close to lasso). As expected, the ridge-like elastic net performs better than ridge if the signal is sparse (1st scenario) and worse than ridge if the signal is dense (2nd scenario). The results for the lasso-like elastic net are similar to those for the lasso. Indeed, it has previously been found that the elastic net without simultaneous tuning of both penalties can mimic ridge or lasso regression (Waldron *et al.*, 2011).

**Fig. 3.**
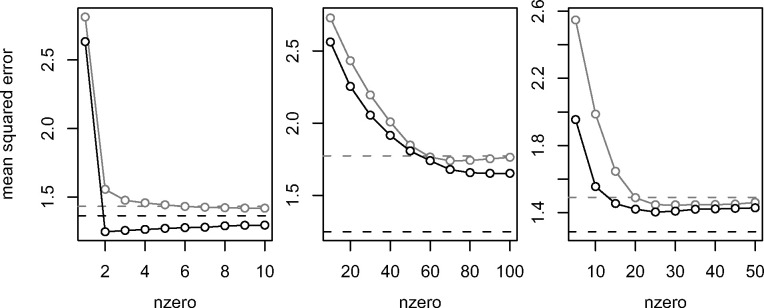
Median out-of-sample mean squared error against number of non-zero coefficients, for the lasso (grey) and the stacked elastic net with *post-hoc* feature selection (black), in the first (left), second (centre), and third (right) scenarios. The dashed lines indicate the medians from the unrestricted versions

Some applications require models with a limited number of selected features. We therefore verified how *post-hoc* feature selection affects the predictive performance of the stacked elastic net. Figure 3 shows the generalization error for different numbers of non-zero coefficients. Models with many selected features tend to be more predictive than models with few selected features. While the stacked elastic net outperforms the lasso given a small number of non-zero coefficients, this difference vanishes for large numbers of non-zero coefficients. *Post-hoc* feature selection increases predictivity if the signal is sparse (1st scenario) and otherwise decreases predictivity (2nd and 3rd scenarios).

### 3.2 Estimation accuracy

If we knew the effects of the features on the outcome, we could not only examine the prediction accuracy but also the estimation accuracy of the stacked elastic net. We adapted the simulation study to make this possible: (i) Simulating the features (*n *×* p* matrix ***X***) from the multivariate Gaussian distribution N(μ,Σ) with a constant mean and correlation structure, namely $\mu_{j}=0,\;\Sigma_{jj}=1$ and $\Sigma_{jk}=0.1$ for all *j* and k≠j in {1,…,p}. (ii) Generating the effects (p×1 vector β) by setting most coefficients to zero and some coefficients to one, namely 5 (sparse scenario), 50 (dense scenario) or 20 (mixed scenario). (iii) Obtaining the outcome (n×1 vector ***y***) by summing up the linear predictor and the residuals (y=Xβ+ε), where the residuals are Gaussian noise with half the (sample) standard deviation of the linear predictor.

In each scenario, we simulated 100 times the feature matrix ***X***, the coefficient vector β, and the outcome vector ***y***, for 100 training and validation samples (but no testing samples). We measure the difference between the true coefficients β and the estimated coefficients $\hat{\beta }$ with the mean absolute error and the mean squared error. For true coefficients equal to zero, stacking is less accurate than tuning. This matches our expectations because stacking leads to denser models than tuning. For true coefficients different from zero, however, stacking is more accurate than tuning. The median decrease in mean absolute error (mean squared error) is 30.4% (15.4%) in the sparse scenario, 3.1% (4.6%) in the dense scenario and 0.4% (1.1%) in the mixed scenario. Stacking is significantly more accurate than tuning in the sparse and dense scenarios in terms of both metrics, according to the two-sided Wilcoxon signed-rank test at the Bonferroni-adjusted 5% level (*P*-value ≤0.05/3).

Additionally, we also examined the selection accuracy. We allowed the stacked elastic with *post-hoc* feature selection, the lasso and the lasso-like elastic net (α=0.95) to include at most 10 features in the model. To compare the selection accuracy, we calculate the precision TP/(TP+FP), where $\text{TP}=\sum_{j=1}^{p}I[{\hat{\beta }}_{j}\neq 0\cap \beta_{j}\neq 0]$ and $\text{FP}=\sum_{j=1}^{p}I[{\hat{\beta }}_{j}\neq 0\cap \beta_{j}=0]$, with TP+FP≤10. Compared to the lasso, the stacked elastic net selects more features among those with an effect ($\bar{\text{TP}}$: 4.4>3.7), and less features among those without an effect ($\bar{\text{FP}}$: 5.2<6.1). Accordingly, the stacked elastic net has a higher mean precision than the lasso in the sparse (57%>51%), dense (36%>27%) and mixed (49%>36%) scenarios. The lasso-like elastic net performs slightly worse than the lasso.

## 4 Application

### 4.1 Benchmark datasets

To further examine the performance of the stacked elastic net, we analysed experimental genomics data. The R package plsgenomics includes three preprocessed gene expression sets for binary or multinomial classification, namely tumour against normal colon tissue (Alon *et al.*, 1999), two kinds of leukaemia (Golub *et al.*, 1999) and four types of small-blue-round-cell tumours (Khan *et al.*, 2001). For the last, we reduced the multinomial problem to four one-versus-rest binary problems. All three datasets are high-dimensional: the first covers 62 samples and 2000 features, the second covers 38 samples and 3051 features, and the third covers 83 samples and 2308 features. We did not perform any further preprocessing to ensure reproducibility and comparability. To obtain robust and almost unbiased estimates of the predictive performance, we used repeated nested cross-validation with 10 repetitions, 10 external folds and 10 internal folds. Table 2 shows the median cross-validated logistic deviance for the six binary classification problems. The stacked elastic net decreases the loss, as compared to ridge, lasso and tuning, except for lasso on the colon dataset. Under *post-hoc* feature selection with the number of non-zero coefficients determined by cross-validation, stacking remains competitive.

**Table 2.** Median cross-validated logistic deviance for three classification problems (rows) under different regularization methods (columns), with the class frequencies (0/1) in the first two columns, and the results for *post-hoc* feature selection in parentheses

	#0	#1	Ridge	Lasso	Tune	Stack	
Colon	22	40	*0.900*	0.820	0.878	0.848	(0.840)
Leukaemia	27	11	*0.252*	0.199	0.145	0.039	(0.165)
SRBCT1	54	29	*0.369*	0.164	0.111	0.078	(0.140)
SRBCT2	72	11	*0.111*	0.035	0.047	0.001	(0.007)
SRBCT3	65	18	*0.258*	0.052	0.052	0.004	(0.005)
SRBCT4	58	25	*0.338*	0.102	0.070	0.015	(0.070)

**Fig. 4.**
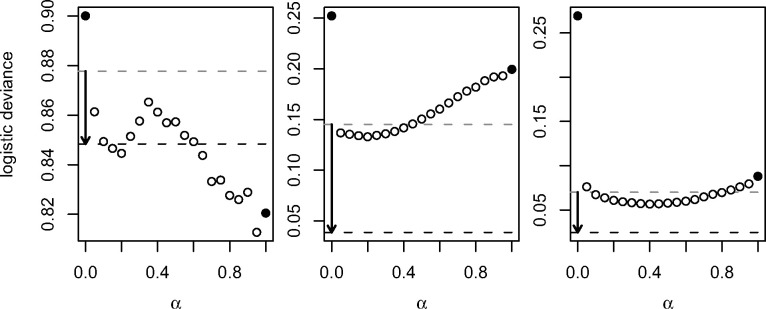
Median cross-validated logistic deviance against the elastic net mixing parameter, for ‘colon’ (left), ‘leukaemia’ (centre), and ‘SRBCT’ (right). The filled circles indicate ridge (α=0) and lasso (α=1) regularization. The dashed lines indicate tuning (grey) and stacking (black). (For ‘SRBCT’, we show the mean over the medians from the four binary problems)

Figure 4 shows the median cross-validated loss for different elastic net mixing parameters. For ‘leukaemia’ and ‘SRBCT’, the loss decreases between 0 (ridge) and some α, and then increases between this α and 1 (lasso). The optimal elastic net mixing parameter, across all cross-validation repetitions, is α=0.95 for ‘colon’, α=0.2 for ‘leukaemia’ and α=0.4 for ‘SRBCT’. If we had known these values before the analysis, we would have minimized the cross-validated loss. Searching for the optimal α in each cross-validation iteration, we either find or miss the optimal α. This is why the tuned elastic net never outperforms the elastic net with the optimal α for a single split. In contrast, the stacked elastic net may outperform the elastic net with the optimal α. We observe this for two out of three applications, namely ‘leukaemia’ and ‘SRBCT’.

### 4.2 Normal/tumour classification

The Cancer Genome Atlas (The Cancer Genome Atlas Research Network *et al.*, 2013) provides genomic data for 33 cancer types. We retrieved the upper quartile normalized RSEM (RNA-Seq by expectation-maximization) TPM (transcript per million) gene expression values (R package curatedTCGAData), merged replicated measurements (R package MultiAssayExperiment) and extracted the sample definitions from the barcodes (R package TCGAutils). We retained ‘solid tissue normal’ (collected near the tumour) and ‘primary solid tumour’ samples. For each cancer type, we retained the 2000 most variably expressed genes, and standardized their expression values.

For cancer types with at least five normal and five tumour samples, we repeatedly trained and validated models with approximately 90% of the samples, and tested the models with approximately 10% of the samples. Table 3 shows the cross-validated logistic deviance under different regularization methods. Here, lasso performs better than ridge for 13 out of 15 cancer types, and stacking performs better than tuning for 11 out of 15 cancer types. The mean decrease in cross-validated logistic deviance from tuning to stacking is 7.5%, and the two-sided Wilcoxon signed-rank test returns a *P*-value of 0.06. *Post-hoc* feature selection with the number of non-zero coefficients determined by cross-validation leads to competitive models, except for cholangiocarcinoma (CHOL). The problem with this cancer type might be the small sample size together with the fact that normal and tumour samples are derived from the same patients. In any case, results for such small sample sizes are inherently unreliable.

**Table 3.** Cross-validated logistic deviance for binary classification problems (rows) under different regularization methods (columns), with the class frequencies (0/1) in the first two columns, and the results for *post-hoc* feature selection in parentheses

	#0	#1	Ridge	Lasso	Tune	Stack	
BLCA	19	389	0.242	0.248	0.265	0.240	(0.240)
BRCA	112	977	0.271	0.197	0.187	0.266	(0.276)
CHOL	9	27	0.754	0.069	0.061	0.023	(0.645)
ESCA	11	172	0.394	0.296	0.302	0.259	(0.260)
HNSC	44	475	0.279	0.291	0.264	0.267	(0.269)
KICH	25	41	0.853	0.755	0.608	0.581	(0.584)
KIRC	72	460	0.198	0.155	0.162	0.159	(0.159)
KIRP	32	257	0.390	0.250	0.299	0.255	(0.252)
LIHC	50	321	0.362	0.330	0.304	0.323	(0.330)
LUAD	59	457	0.262	0.245	0.253	0.206	(0.203)
LUSC	51	450	0.161	0.076	0.078	0.073	(0.073)
PRAD	52	444	0.444	0.438	0.464	0.442	(0.488)
STAD	35	383	0.339	0.237	0.230	0.237	(0.237)
THCA	59	434	0.431	0.393	0.463	0.458	(0.459)
UCEC	10	360	0.088	0.072	0.085	0.062	(0.059)

## 5 Discussion

The elastic net is the method of choice for many biomedical applications, because it renders predictive and interpretable models. It weights between ridge and lasso regularization, but the optimal weighting is often unknown. Instead of selecting one weighting by tuning, we combine multiple weightings by stacking. According to our empirical analyses, this improves the predictive performance of the elastic net in various settings. The increase in computational cost is negligible, because the only addition is the low-dimensional regression of the outcome on the cross-validated linear predictors. The equivalence between stacking linear predictors and pooling regression coefficients allows us to increase the predictive performance while maintaining the interpretability of the regression coefficients.

In contrast to the lasso, the stacked elastic net might or might not perform feature selection. It selects features unless the meta learner includes the base learner with pure ridge regularization, but it tends to select more features than the tuned elastic net, because it combines multiple base learners. The stacked elastic net selects a feature if and only if the meta learner selects a base learner that selects this feature. It is therefore possible to impose feature selection by excluding the base learner with pure ridge regularization (α>0). As this might fail to render sufficiently sparse models, we suggest to perform *post-hoc* feature selection (Hahn and Carvalho, 2015) but recommend to verify by cross-validation whether imposing sparsity makes the model much less predictive.

An extension of the stacked elastic net would be to use a fused penalty (Tibshirani *et al.*, 2005) for the meta learner, because the base learners are related in regard to the elastic net mixing parameter. Another extension would be to combine two ensemble techniques, namely stacking and bagging. While stacking involves fitting different models to the same samples and *weighting* the predictions, bagging involves fitting the same model to different bootstrap samples and *averaging* the predictions. Since random (bagged) regressions seem to be competitive with random forests (Song *et al.*, 2013), we could potentially combine stacking and bagging to make elastic net regression even more predictive without making it less interpretable.
